# Unilateral Maxillary First Molar Extraction in Class II Subdivision: An Unconventional Treatment Alternative

**DOI:** 10.1155/2016/2168367

**Published:** 2016-04-21

**Authors:** J. W. Booij, Christos Livas

**Affiliations:** ^1^Private Practice, Schelluinsevliet 5, 4203 NB Gorinchem, Netherlands; ^2^Department of Orthodontics, University of Groningen, University Medical Center Groningen, Hanzeplein 1, Triadegebouw, Ingang 24, 9700 RB Groningen, Netherlands

## Abstract

The asymmetrical intra-arch relationship in Class II subdivision malocclusion poses challenges in the treatment planning and mechanotherapy of such cases. This case report demonstrates a treatment technique engaging unilateral extraction of a maxillary first molar and Begg fixed appliances. The outcome stability and the enhancing effect on the eruption of the third molar in the extraction segment were confirmed by a 4-year follow-up examination.

## 1. Introduction

Class II subdivision malocclusion is a dentofacial deformity, estimated to account for up to 50% of Class II malocclusions [1]. It possesses characteristics of both Class I and Class II malocclusion resulting in asymmetry between the right and the left sides of the dentition. Depending on the location of asymmetry, unilateral mechanics is performed to achieve distalization of the mesially positioned maxillary first molar or protraction of the opposing segment. Asymmetrical headgear, coil springs coupled with Class II elastics or TADs [2], fixed functional appliances [3], or asymmetrical premolar extraction patterns [4, 5] are commonly applied in growing patients to correct the Class II occlusion on the affected side.

A less typical treatment strategy combining single extraction of a maxillary first molar and Begg light-wire appliances showed favourable outcomes in terms of occlusion, facial profile, and midline esthetics on average in 2.5 years after appliance removal [6].

This case report describes the orthodontic management of a Class II subdivision patient treated with the abovementioned protocol.

## 2. Case Report

**Figure 1:**
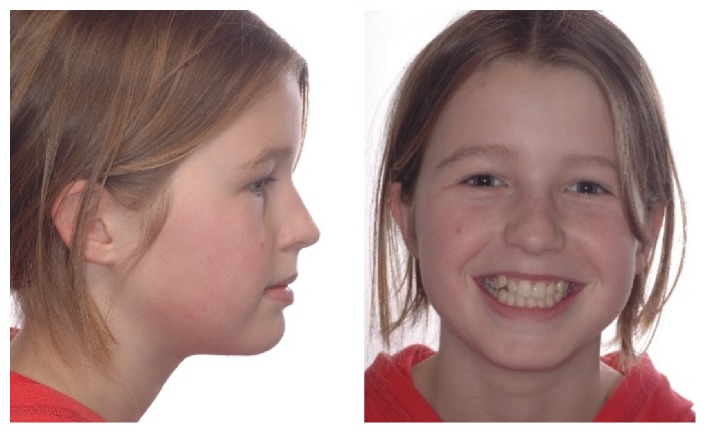
Pretreatment extraoral photographs.

**Figure 2:**
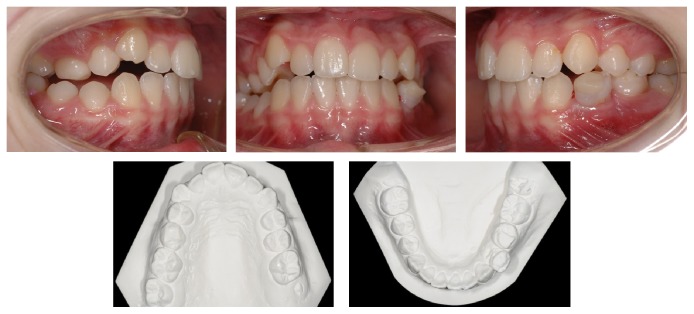
Pretreatment intraoral photographs and study casts (occlusal view).

**Figure 3:**
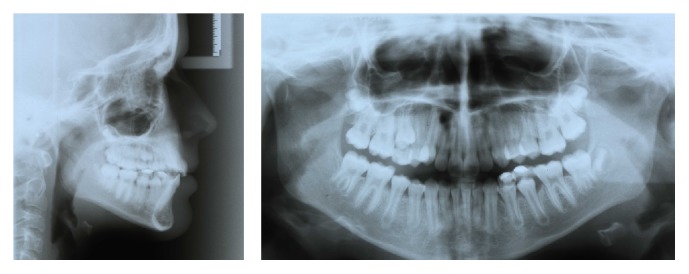
Pretreatment radiographs.

A 14-year-old female was diagnosed with Class II subdivision malocclusion on the right side and a maxillary-to-facial midline discrepancy of 2 mm (Figures 1 and 2). During the intake, the patient expressed her concerns in complying with extraoral anchorage devices, cumbersome orthodontic accessories, or intermaxillary elastics for a long period. Clinical examination revealed fully erupted maxillary second molars and persistent 55, 74, and 75. With the exception of 48, no tooth agenesis was confirmed by the orthopantomogram (Figure 3). To meet the patient's demands, extraction of the right maxillary first molar was proposed instead.

**Figure 4:**
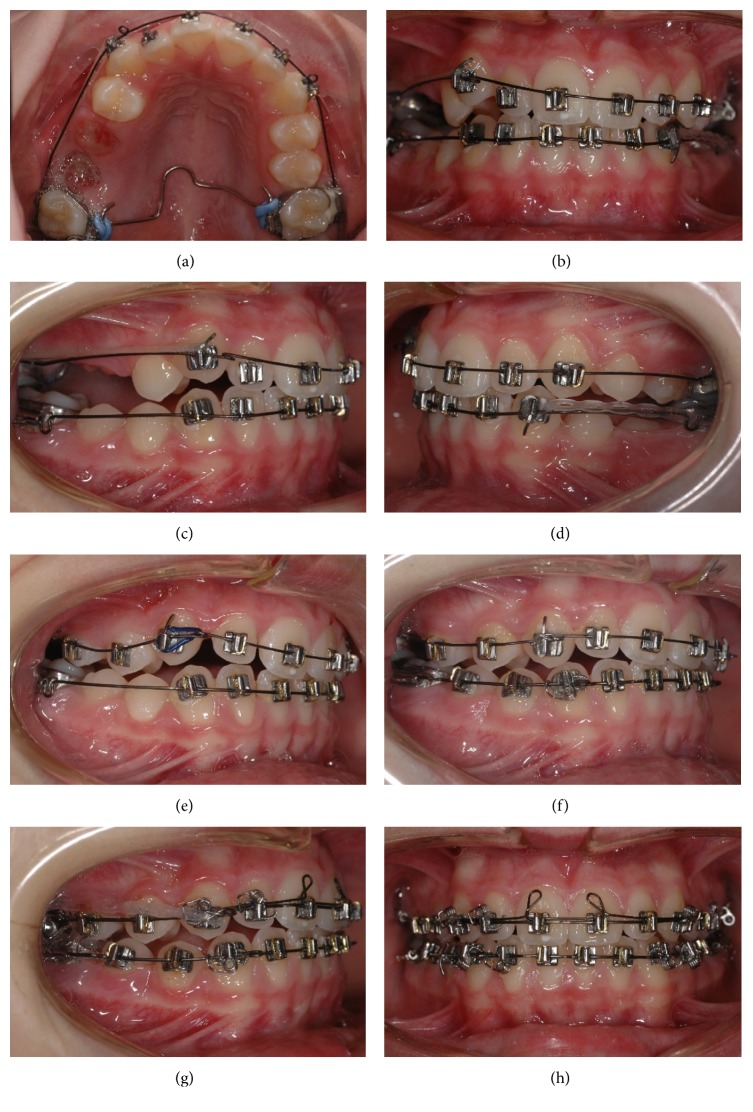
(a–d) Class II correction on the right side using TPA anchorage and horizontal elastics. In this phase, premolars were not bonded to facilitate sliding mechanics. (e-f) After achieving Class I premolar relationship, the remaining teeth were bonded. (g) Space closure with elastic power chain. (g, h) Torque correction by means of a customized two-spur torque auxiliary of 0.014-inch regular wire and uprighting springs on the maxillary canine brackets.

Before extracting 16 and persistent deciduous molars, bands with 6 mm single 0.022-inch round buccal tubes and palatal sheaths were placed on 17 and 26. After a healing period of 3 weeks, Begg brackets were placed on the anterior maxillary and mandibular teeth. To prevent second molar rotation, a transpalatal arch (TPA) was inserted. Second molar anchorage was reinforced by anchor bends on a customized 0.016-inch premium plus pull-straightened Australian archwire (Wilcock, Whittlesea, Australia) mesial of the molar tube to counteract unwanted mesial movement of 16 into the extraction space (Figure 4(a)). High hat lock pins (TP Orthodontics, Westville, IN, USA) were placed on maxillary canines and partially bent mesially to receive light 8 mm horizontal elastics (5/16 inches) on the Class II buccal segment extending to the buccal hook on the maxillary second molar band (Figures 4(a)–4(c)). The patient was instructed to replace the Class I elastics on a weekly basis. By bending circle-shaped loops mesial to the canine brackets, controlled retraction of the anterior teeth was achieved. Visits were scheduled 6- to 8-week intervals. The initially malpositioned 12 was engaged to the archwire until adequate space had been created by canine distalization (Figures 4(b) and 4(c)). After 6 months, Class I premolar occlusion was achieved, the premolars were also bonded with light-wire brackets, and Class II elastic wear was instructed for night-time. After alignment of the maxillary premolars, the 0.016-inch starting wire was replaced by a 0.018-inch premium plus archwire (Wilcock). Additionally, an individual two-spur torque auxiliary of 0.014-inch regular wire (Wilcock) was inserted in the anterior maxillary region to produce proper palatal root torque. For the same reason, uprighting springs (TP, La Porte, Indiana, USA) were fixed in the vertical slots of the canine brackets (Figures 4(g) and 4(h)). Closure of the residual extraction spaces in the maxillary right buccal segment was carried out with elastic power chains. In the final treatment stage, adjustments were made in the archwires and uprighting springs independently for each tooth for detailed finishing. Completing treatment, canine-to-canine retainers made of multistranded wire were bonded in both arches.

**Figure 5:**
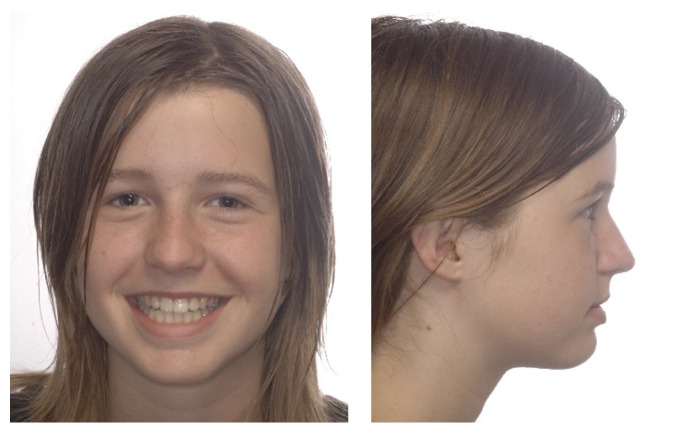
Posttreatment extraoral photographs.

**Figure 6:**
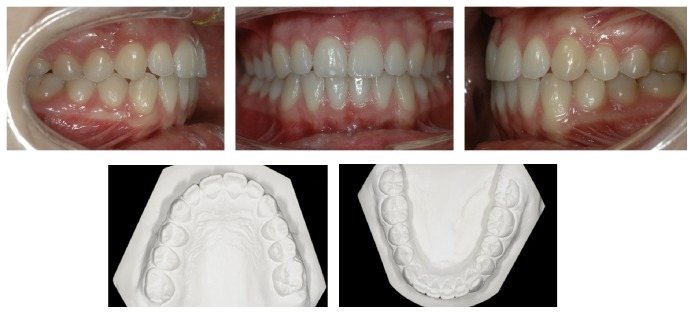
Posttreatment intraoral photographs and study casts (occlusal view).

**Figure 7:**
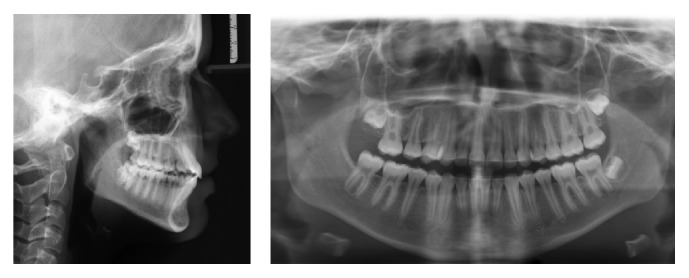
Posttreatment radiographs.

**Figure 8:**
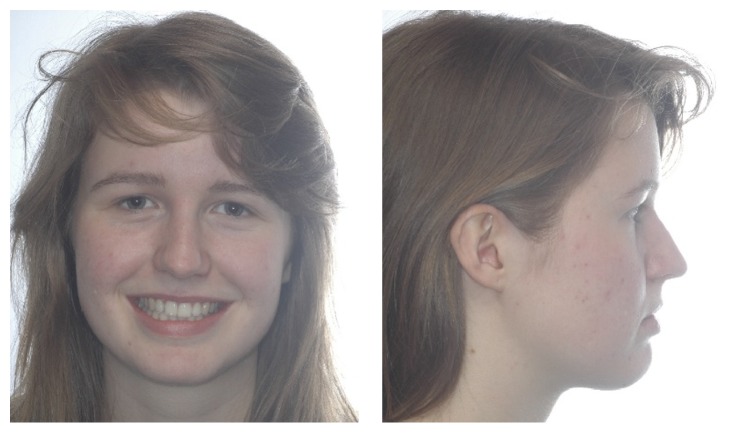
Four-year follow-up extraoral photographs.

**Figure 9:**
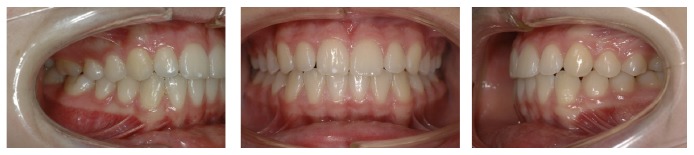
Four-year follow-up intraoral photographs.

**Figure 10:**
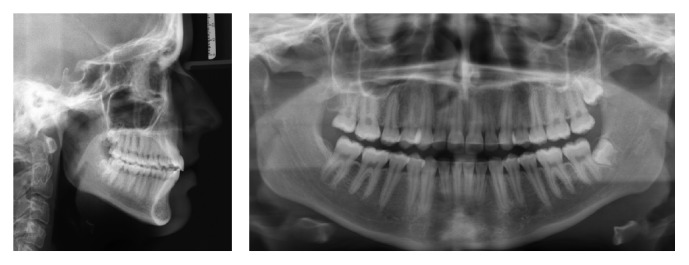
Four-year follow-up radiographs.

The active treatment lasted 26 months. Class I occlusion, tooth alignment, and midline correction were maintained for 4 years after appliance removal (Figures 6 and 9). Anterior tooth retraction did not compromise the soft tissue profile (Figures 5 and 8). Eruption of 18 was accelerated reaching occlusal contact with the antagonist, while the contralateral molar remained unerupted (Figures 7 and 10).

## 3. Discussion

Our Class II subdivision technique led to good occlusal and esthetic outcomes, which were preserved for 4 years after active treatment had been completed. Besides stable end results in the long term [6], a positive effect on the axial inclination of maxillary third molars was demonstrated in Class II subdivision cases treated with unilateral maxillary first molar extraction and low friction fixed appliances [7]. Maxillary third molars in the extraction side became 3.1–3.4 times more upright than the contralateral teeth [7]. Likewise in this case report, eruption of the maxillary third molar in the extraction segment was strikingly enhanced.

Patient cooperation was restricted to oral hygiene measures and once-per-week replacement of elastics, which may render this method suitable for patients with poor compliance [8]. Modification of this treatment method with bilateral extraction of maxillary first molars has been previously described as “less-compliance therapy” [8].

Longer treatment duration has been observed in asymmetric premolar extraction protocols [4, 5] compared to orthodontic therapy with either unilateral maxillary first molar extraction [6] or Herbst and fixed appliances [3]. Nonetheless, with respect to the end molar occlusion, Class III in the original Class I side may be expected in Class II subdivision patients treated with fixed functional appliances [3].

Without doubt, the popularity of Begg or similar techniques declined dramatically during the last 30 years [9]. However, orthodontic mechanics including application of light elastic forces, anchorage bends, or delayed bonding of premolars during space closure may be integrated in the philosophy of contemporary straight-wire techniques.

Premolar extraction schemes are prescribed by orthodontists in the United States in 85% of extraction cases [9]. From the ethical point of view, a decision to electively extract healthy premolar teeth for orthodontic purposes may not be warranted in cases with compromised first molars. As a general rule, presence of extensive caries lesions, large restorations, endodontic or periodontal problems, or hypoplastic enamel should be taken into account when extraction treatment has been chosen. The first permanent molar has the shortest caries-free survival under the age of 8 years [10]. It also represents the most caries prone tooth in children older than 11 years [11]. In addition to this, first molars can suffer from developmental enamel hypomineralisation of unknown aetiology often affecting permanent incisors. Lately published rates vary between 4.2 and 21.4% depending on the country and examination method [12–14]. Prognosis of endodontics in multirooted teeth may be also questionable. In this context, the first molar has been reported as the most commonly extracted tooth due to endodontic complications [15]. Under such circumstances and in presence of fully erupted maxillary second molars, well-formed third molar at the Class II buccal segment, maxillary dental asymmetry, and fairly aligned mandibular arch, extraction of a maxillary first molar may be a viable option in treating asymmetrical Class II malocclusion cases.

## 4. Conclusion

This 4-year follow-up case report indicates that unilateral extraction of a maxillary first molar in selected cases might be a rewarding treatment alternative in Class II subdivision subjects and especially in those with compliance issues.
